# Advanced 3D Face Reconstruction from Single 2D Images Using Enhanced Adversarial Neural Networks and Graph Neural Networks

**DOI:** 10.3390/s24196280

**Published:** 2024-09-28

**Authors:** Mohamed Fathallah, Sherif Eletriby, Maazen Alsabaan, Mohamed I. Ibrahem, Gamal Farok

**Affiliations:** 1 Department of Computer Science, Faculty of Computers and Information, Kafr El-Sheikh University, Kafr El-Sheikh 33511, Egypt; 2 Department of Computer Science, Faculty of Computers and Information, Menoufia University, Menoufia 32511, Egypt; sherif.eletriby@ci.menofia.edu.eg (S.E.); gamal.farouk@ci.menofia.edu.eg (G.F.); 3 Department of Computer Engineering, College of Computer and Information Sciences, King Saud University, P.O. Box 51178, Riyadh 11543, Saudi Arabia; malsabaan@ksu.edu.sa; 4 School of Computer and Cyber Sciences, Augusta University, Augusta, GA 30912, USA; mibrahem@augusta.edu

**Keywords:** GANs, 3D reconstruction, GCNs, efficient-net

## Abstract

This paper presents a novel framework for 3D face reconstruction from single 2D images and addresses critical limitations in existing methods. Our approach integrates modified adversarial neural networks with graph neural networks to achieve state-of-the-art performance. Key innovations include (1) a generator architecture based on Graph Convolutional Networks (GCNs) with a novel loss function and identity blocks, mitigating mode collapse and instability; (2) the integration of facial landmarks and a non-parametric efficient-net decoder for enhanced feature capture; and (3) a lightweight GCN-based discriminator for improved accuracy and stability. Evaluated on the 300W-LP and AFLW2000-3D datasets, our method outperforms existing approaches, reducing Chamfer Distance by 62.7% and Earth Mover’s Distance by 57.1% on 300W-LP. Moreover, our framework demonstrates superior robustness to variations in head positioning, occlusion, noise, and lighting conditions while achieving significantly faster processing times.

## 1. Introduction

The field of 3D face reconstruction from 2D images has seen significant advancements in recent years, driven by the increasing demand for accurate and efficient 3D modelling in various applications such as virtual reality, gaming, and biometric authentication. However, existing methods often struggle with challenges such as limited facial detail capture, instability in training, and difficulty in handling diverse facial poses and expressions.

Our research is motivated by the need to overcome these limitations and provide a more robust and versatile solution for 3D face reconstruction. We propose a novel approach that leverages the strengths of both adversarial generative networks (GANs) and graph neural networks (GNNs) to achieve this goal.

The use of GANs in our framework is motivated by their proven capability to generate highly realistic and detailed images. In the context of 3D face reconstruction, GANs can help in capturing fine-grained facial features and textures that might be lost in traditional reconstruction methods. However, GANs are known to suffer from issues such as mode collapse and training instability, which we address through our modified architecture and training process.

Graph neural networks, on the other hand, are particularly well-suited for processing 3D mesh data due to their ability to operate directly on graph-structured data. By incorporating GNNs into our model, we can more effectively capture the spatial relationships between facial landmarks and mesh vertices, leading to more accurate geometric reconstructions. This is especially beneficial when dealing with complex facial topologies and varying expressions.

Research on reconstructing 3D facial models from 2D images, depth images, and 3D scans is currently a hot research topic. Advancements in deep neural network training for supervised learning have led to significant advances in 3D face reconstruction from a single 2D image. Blanz and Vetter [1] introduced the 3D Morphable Model (3DMM), which served as the foundation for this reconstruction process. It employs principal component analysis (PCA) to capture variations in facial shape, texture, and expressions, enabling the generation of realistic 3D facial representations by optimizing coefficients using methods like gradient descent. The 3DMM effectively translates 2D image information into detailed 3D models, making it a pivotal tool in facial analysis, animation, and biometric applications [1,2,3,4,5].

The use of deep neural networks has revolutionized the field of 3D facial reconstruction. This technique involves using a convolutional neural network (CNN) for facial image analysis, generation, and approximation of the 3DMM coefficients. This technique has significantly enhanced the accuracy and efficiency of 3D facial reconstruction processes, marking a substantial advancement in this domain [6,7,8]. Initially, a trained CNN extracts facial features from images. Subsequently, it utilizes these features to estimate 3DMM coefficients, leading to a more precise reconstruction of a 3D facial model. Notably, CNN-based methods excel in swiftly and accurately estimating 3DMM coefficients, resulting in efficient and precise 3D face reconstructions. CNN has the limitation of only working with 2D grid-structure images. The 3D mesh models have a graph structure (vertices and edges), where each image or 3D model can have a different input shape. Graph neural networks (GNNs) [9] and graph convolutional networks (GCNs) [10] are frequently employed in neural networks to manage graph structure data. These networks execute operations on these structures akin to convolutional operations performed on 2D images, offering a robust framework for graph-based computations in deep learning architectures.

Many papers have utilized GANs as the foundational model for generating 3D images, yet they often overlook the common challenges associated with GAN training, such as mode collapse, superior discriminator, and unstable training [7,11]. The issue of mode collapse has been widely observed in GAN training [12,13,14], wherein the generator becomes fixated on specific distributions (modes) of the real data. These modes represent samples of data that the discriminator consistently recognizes as authentic distributions. Consequently, the collapsed discriminator provides meaningless feedback to the generator, leading it to repeatedly generate the same data distributions (modes). This phenomenon restricts the generator’s sampling to a small subset of the real data, causing the GAN to collapse into these limited distributions. As a result, the generator tends to produce identical or similar images, reducing the diversity and quality of the generated content.

This research presents an innovative technique that uses generative neural networks to generate a 3D mesh model from a single 2D image in addition to its landmark. Our generator and discriminator architectures include graph convolution layers. The model based on the IGAN [15] technique, focuses on the quality of the produced images as well as the model’s stability during training. Our technique focuses on using the landmarks as input to the generator, resolving mode collapse, and stabilizing the generator architecture. We use a unified branch in our model to create face geometry, emotion, and positioning. The generator creates a three-dimensional facial geometry mesh model. In comparison to a reference item, our model performs well at reconstructing facial expressions.

Our contribution is outlined as follows:

1. Proposes a novel method for reconstructing facial geometry from a single 2D image and its landmarks, offering faster processing times and robustness to variations in head positioning, occlusion, noise, and lighting, making it suitable for real-time applications.
2. Introduces a GCN-based generator architecture with custom identity blocks and a modified loss function, which effectively mitigates mode collapse, increases training speed, and reduces the training instability commonly encountered in GANs.
3. Develops a lightweight GCN-based discriminator that enhances both the accuracy and stability of the generated 3D facial models, directly contributing to faster and more efficient processing.
4. Achieves state-of-the-art performance on benchmark datasets, with significant improvements in Chamfer Distance, Cross Entropy loss, and Earth Mover’s Distance metrics, further validating the method’s accuracy, stability, and computational efficiency.

## 2. Related Work

This section provides an overview of key developments in 3D facial reconstruction, organized into three main categories: 3DMM-based methods, deep learning approaches, and GAN-based techniques.

The field of 3D facial reconstruction has seen significant advancements since the seminal work of Blanz and Vetter [1], who introduced the 3D Morphable Model (3DMM). The 3DMM, a linear model leveraging principal component analysis (PCA), facilitates the dimensionality reduction of 3D face model datasets. This enables the independent manipulation of facial shape, texture, and expression parameters. 3DMM-based approaches offer stable linear models and good reconstruction performance. However, as they are linear models, they have typical issues, such as challenges in reconstructing faces that differ from the data range used to produce the 3DMM and dealing with fine details like wrinkles and wearable objects.

The introduction of deep learning has revolutionized 3D reconstruction research, shifting focus towards directly estimating 3DMM coefficients from 2D images [16,17,18]. Recognizing the limitations of pixel-wise error metrics in achieving optimal 3D facial reconstructions, Deng et al. [19] proposed a novel approach that leverages unsupervised and semi-supervised learning. They argue that relying solely on pixel-wise comparisons between the original image and the rendered model can lead to convergence on local minima, potentially hindering reconstruction accuracy. To address this, their approach incorporates a pre-trained facial recognition network. This network extracts feature vectors from both the original and reconstructed faces. The cosine distance between these feature vectors is then employed as the error metric, guiding the reconstruction process towards a more semantically meaningful solution.

Graph Neural Networks (GNNs) have emerged as a powerful tool for processing data with inherent graph-like structures, making them particularly suitable for 3D face reconstruction tasks. Unlike traditional neural networks that operate on regular grid-like data (e.g., images), GNNs can directly process and learn from graph-structured data, such as 3D meshes. Scarselli et al. [20] introduced the foundational concept of GNNs, which has since been extended and refined. A key development in this field was the introduction of Graph Convolutional Networks (GCNs) by Defferrard et al. [10], which efficiently perform convolution operations on graph-structured data in the context of 3D face reconstruction.

Another significant approach for 3D reconstruction is based on generative adversarial networks (GANs) [7,21]. Gecer et al. [7] tried to overcome the limitation of 3DMMs in capturing high-frequency facial texture details during reconstruction. They addressed this challenge by introducing GANs into the reconstruction pipeline. The trained GAN effectively extracted high-resolution face textures with high-frequency components from input photos. Our method builds on this by incorporating a GAN to generate realistic and detailed textures. However, our approach goes a step further by leveraging modified graph neural networks (GNNs). This is motivated by the inherent strengths of GNNs in modelling relationships between facial landmarks. By incorporating GNNs, we aim to capture the intricate spatial dependencies within the facial structure, leading to more accurate and nuanced reconstructions compared to solely relying on 3DMMs or GANs alone. Ref. [21] introduces GAN2Shape, a novel generative neural network. The GAN was trained on 2D images only and was able to generate 3D face images, but the quality was poor due to difficulties in estimating the position of objects and a lack of landmarks.

Several recent studies have explored alternative approaches to 3D facial reconstruction that bypass the use of 3DMMs and traditional GAN approaches. These methods directly predict the 3D coordinates or texture of each vertex in the model using GCNs, demonstrating promise for achieving more detailed reconstructions. Lin et al. [20] pioneered this approach for fine-grained texture reconstruction. Their method utilizes GCNs within a dedicated texture refinement module, consisting of three distinct GCNs categorized as decoders, refiners, and combiners. The decoder GCN leverages feature vectors extracted from a pre-trained facial recognition network (e.g., FaceNet [22]) to generate detailed textures. To improve its quality, the refiner GCN uses the initial coarse texture obtained from a traditional 3DMM method. Finally, the combiner GCN integrates the outputs from both decoders and refiners to produce the final vertex color, resulting in fine-grained textures. Subsequent adversarial training with a discriminator further refines the results.

Cheng et al. [23] introduced a novel approach for detailed facial reconstruction. Their method leverages a divide-and-conquer strategy, employing separate networks for reconstructing coarse and fine facial geometry. Notably, to capture detailed shapes, they incorporate a Graph Convolutional Network (GCN) refiner with a shape-from-shading (SfS) loss [24]. This refiner network injects feature vectors obtained during the CNN encoder process, ensuring that information from the input image is preserved. In a complementary approach, Deng et al. [25] proposed an effective facial reconstruction method that utilizes an attention mechanism within a Convolutional Neural Network (CNN) framework. This attention mechanism focuses on the feature vectors extracted from the input image, effectively suppressing background noise and enhancing the prominence of facial features. Consequently, this method contributes to improved facial reconstruction accuracy.

Zhou et al. [26] proposed an encoder-decoder architecture that integrates CNNs and GCNs for simultaneous facial shape and texture reconstruction to address the challenges in 3DMM models, such as linearity. While the aforementioned methods have shown promising results in 3D face reconstruction, they face several limitations that our GNN-based approach addresses. Traditional CNNs used in many existing methods (e.g., [22,25,26]) operate on regular grid structures, which can struggle to capture the complex spatial relationships in 3D facial geometry and also struggle with stable training. Our GNN-based approach naturally models these spatial relationships through graph structures, allowing for a more accurate reconstruction of facial features and their relative positions. Additionally, many existing methods (e.g., [7,21]) do not explicitly model the structural relationships between facial features. Our approach, by using a graph representation, inherently models these structural relationships, leading to more coherent reconstructions. To mitigate these issues and achieve stable results, our model emphasizes the importance of employing strong regularization techniques following the architecture of PWGAN and IGAN [14,15].

## 3. Materials and Methods

This section presents the methodology for our proposed framework, which generates 3D facial models from a single 2D image. We leverage a combination of advanced techniques, GCNs, GANs, and data augmentation strategies to improve the accuracy, stability, and speed of the reconstruction process. The proposed method is designed to handle variations in head positioning, occlusion, noise, and lighting conditions.

**Figure 1:**
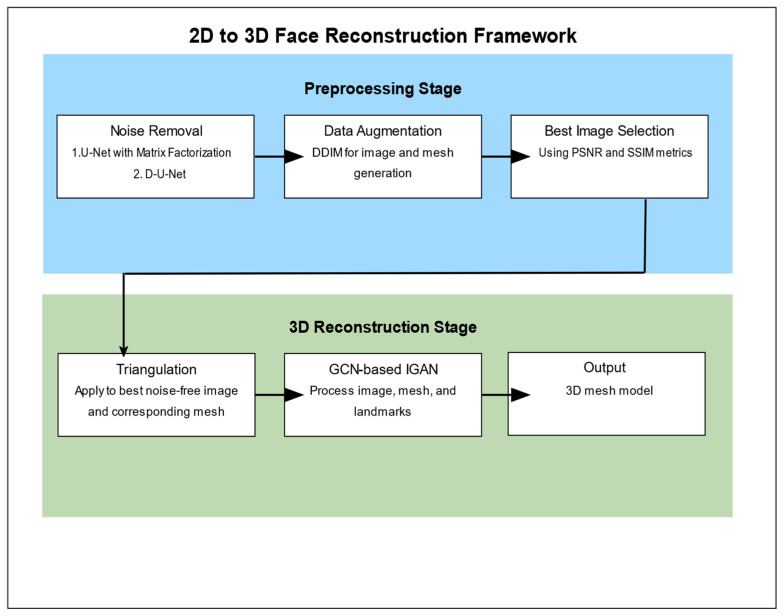
The proposed framework methodology.

The framework consists of two main stages: preprocessing and 3D construction. The preprocessing stage focuses on noise removal and data augmentation while the 3D construction stage applies a GCN-based generative adversarial network to generate the final 3D model. The following subsections detail the key components of our approach, as outlined in Algorithm 1 and Figure 1.

Figure 1 presents a comprehensive overview of our 2D to 3D face reconstruction framework. The process is divided into two main stages: Preprocessing and 3D Reconstruction. The Preprocessing stage consists of three key steps:

1. Noise Removal: We employ two methods—U-Net with Matrix Factorization and D-U-Net—to reduce noise in the input 2D images.
2. Data Augmentation: Using the Denoising Diffusion Implicit Model (DDIM), we generate and edit both 2D images and 3D meshes to enhance our dataset.
3. Best Image Selection: We select the most suitable noise-free image using Peak Signal-to-Noise Ratio (PSNR) and Structural Similarity Index (SSIM) metrics.

The 3D Reconstruction stage involves the following steps:

1. Triangulation: We apply triangulation to the best noise-free image and its corresponding mesh.
2. GCN-based IGAN: Our modified Graph Convolutional Network (GCN)-based Improved Generative Adversarial Network (IGAN) processes the image, mesh, and landmarks.
3. Output: The final result is a detailed 3D mesh model of the face.

**Algorithm 1:** 2D to 3D Reconstruction Using IGAN
**Input:** 2D Face Image.
**Start The Preprocessing Stage****1****Step 1:** Noise Removal**2**
Use U-Net with matrix factorization on image to remove image noise.**4**
Use D-U-Net to remove image noise.**5**
Choose best noise-free image using PSNR and SSIM metrics.**6****Step 2:** Data Augmentation**7**
Use LDM to augment the training dataset.**8**
**FOR EACH** Face Image **DO****9**

Generate multiple augmented images using DDIM or Pix2Pix.**10**

Adding pose change, smile, glasses, etc., to faces (the augmentations).**11**

Generate multiple augmented mesh model with same transformation.**12**

Pair the images and meshes that have same transformation into new samples.**13**
**END FOR****14****Start The 3D Construction Stage****15**Apply triangulation on mesh pair mesh with best Free_Noise_image.**16**Pass the pair to GCN (based which is based IGAN) along with landmarks.**17****Output:** 3D mesh model.

### 3.1. Preprocessing

The preprocessing stage focuses on two main tasks: noise removal and data augmentation. To reduce image noise, we employ U-Net with Matrix Factorization, which effectively improves image quality. The best noise-free images are then selected based on Peak Signal-to-Noise Ratio (PSNR) and Structural Similarity Index (SSIM) metrics, ensuring high-quality inputs for the subsequent 3D reconstruction stage. This noise reduction process is essential for handling common issues like lighting variations and occlusions, leading to more accurate reconstructions.

In addition to noise removal, we apply data augmentation using Denoising Diffusion Implicit Models (DDIM) [27], which is known for generating high-quality synthetic data. This augmentation step enriches our dataset by improving its diversity and robustness, thus enhancing the accuracy of the 3D reconstruction model. In our model, we adopt LDM [28] since it maintains a balance between diversity, fidelity, and speed. Our DDIM can generate and edit the mesh and 2D image based on [29].

### 3.2. Generative Adversarial Networks Modification

Generative adversarial networks have emerged as a powerful framework in deep learning, particularly in image generation tasks. The essence of GANs lies in their unique architecture, which comprises two neural networks, the generator and the discriminator, engaged in an adversarial game. The generator $G\left(z\right)$ aims to produce realistic outputs, such as images, by minimizing the loss function (1), while the discriminator’s D(x) role is to distinguish between real images and generated images by maximizing the loss function (2). Through iterative training, GANs learn to generate increasingly realistic samples by leveraging the feedback loop between the generator’s attempts to fool the discriminator and the discriminator’s improvements in distinguishing real from fake data; therefore, the objective function of GAN is a minmax game, which can be formally defined by Equation (3).

(1) $$L_{G}=-E_{z∼p\left(z\right)}\left[\log D\left(G\left(z\right)\right)\right]$$

(2) $$L_{D}=-E_{x∼p_{\mathrm{data}}\left(x\right)}\left[\log D\left(x\right)\right]-E_{z∼p\left(z\right)}\left[\log\left(1-D\left(G\left(z\right)\right)\right)\right]$$

(3) $$\underset{G}{\min}\underset{D}{\max}V\left(D,G\right)=E_{x∼p_{\mathrm{data}}\left(x\right)}\left[\log D\left(x\right)\right]+E_{z∼p\left(z\right)}\left[\log\left(1-D\left(G\left(z\right)\right)\right)\right]$$

These equations represent the standard formulation of the GAN loss functions, where (*x*) is the discriminator’s output for real data *x*, while G(z) is the generator’s output given. The variable $z\in R^{d_{z}}$ is sampled from a normal distribution $p\left(z\right)∼N\left(0,I\right)$. Here, the zero signifies that the mean of the normal distribution is zero, while I denotes the identity matrix, indicating unit variance across all dimensions. $data\left(x\right)$ is the real data distribution

However, GAN training can face challenges such as mode collapse, where the generator converges to a limited set of outputs, and instability in training, leading to suboptimal results. Strategies like minibatch training and label smoothing have been proposed to address these challenges and improve the stability and diversity of GAN-generated outputs.

We implemented several modifications to the standard GAN architecture, focusing on both the discriminator and generator, to address inherent issues and enhance performance. These modifications serve as the foundation for integrating a Graph Convolutional Network to generate 3D images from a single 2D image.

**Figure 2:**
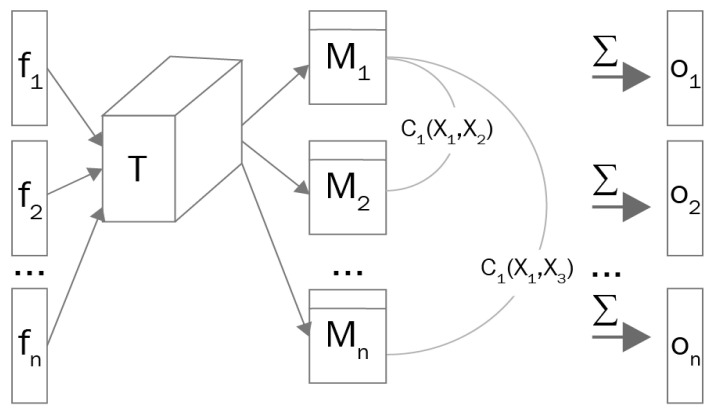
Mini-batch discrimination working mechanism [30].

The first modification involves the use of minibatch training [30] as shown in Figure 2, on the discriminator to address the problem in which the discriminator’s gradients align across various points in the data distribution. Put simply, the generator consistently generates similar output images, irrespective of the latent variable’s sampling location. The implementation of minibatch training allows the discriminator to analyze multiple data instances simultaneously and understand the relationships between them. This approach has proven effective in preventing mode collapse in deep learning [31].

**Figure 3:**
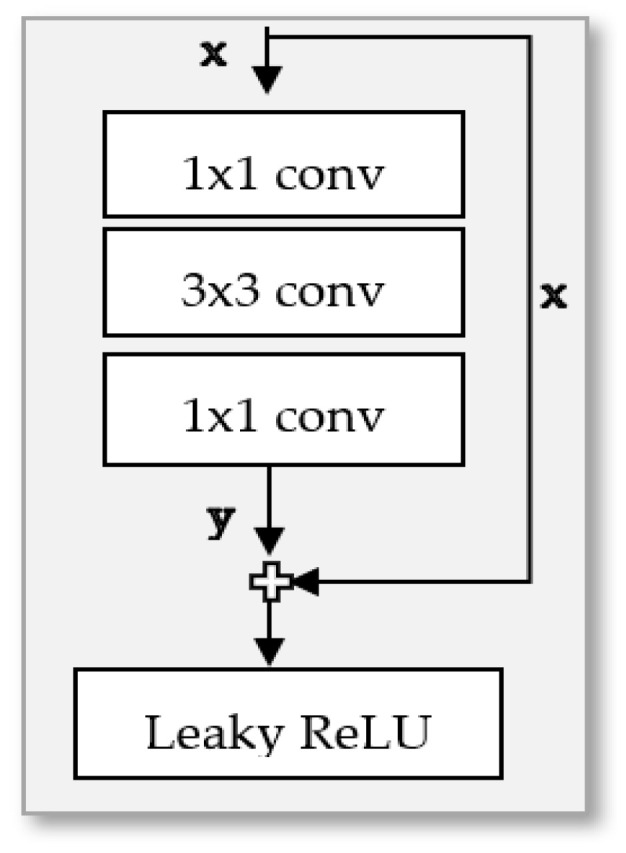
The architecture of identity block [15].

**Figure 4:**
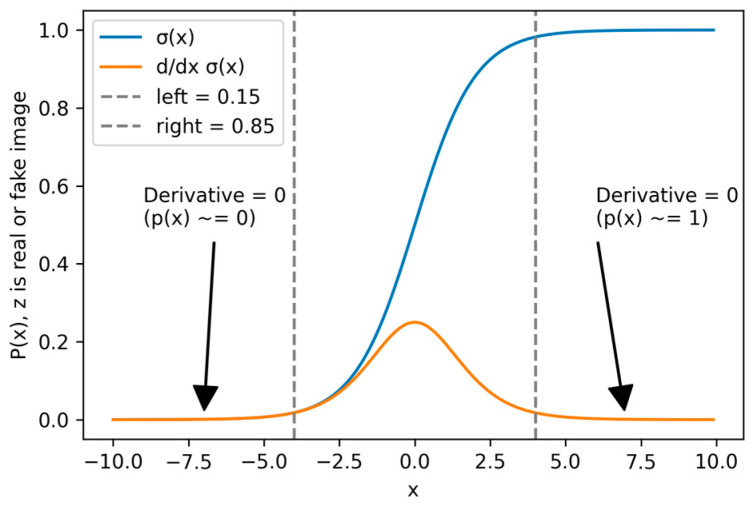
The label smoothing, we use on our GAN approach to solve the problem of vanishing gradient.

We made another modification to the base GAN architecture by incorporating the identity block [15], which is shown in Figure 3, which successfully decreased training duration. In addition to this modification, we smoothed the output of GAN’s discriminator to 0.9 and 0.01 instead of 1 and 0 to solve the vanishing gradient since GAN uses a sigmoid loss function as shown in Figure 4. These enhancements are achievable even with constrained computational resources.

### 3.3. 3D Reconstruction Network Architecture

**Figure 5:**
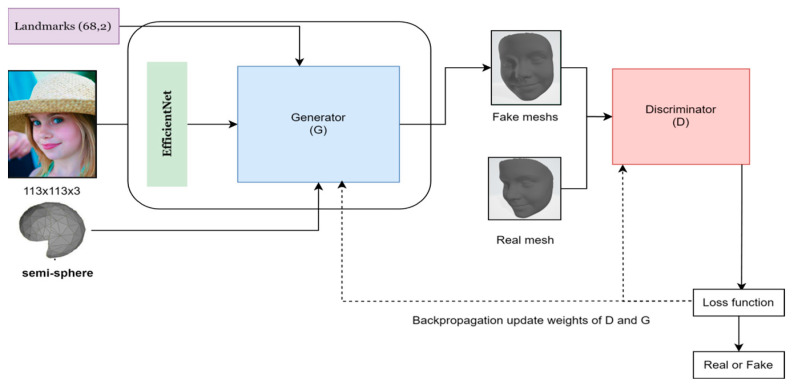
Comprehensive overview of our 3D reconstruction network architecture.

Figure 5 presents a comprehensive overview of our 3D reconstruction network architecture. The network consists of two main components: a generator and a discriminator. The generator takes a 2D input image along with its landmarks and processes it through an EfficientNet encoder, followed by reconstruction blocks, triangulation layers, and GCN layers to produce a 3D mesh model. The discriminator, composed of a convolutional graph layer, an advanced GCN block, a mean node function, and a linear classification layer, evaluates the authenticity of the generated 3D models. In the following subsections, we detail each component of this architecture.

#### 3.3.1. Generator Architecture

**Figure 6:**
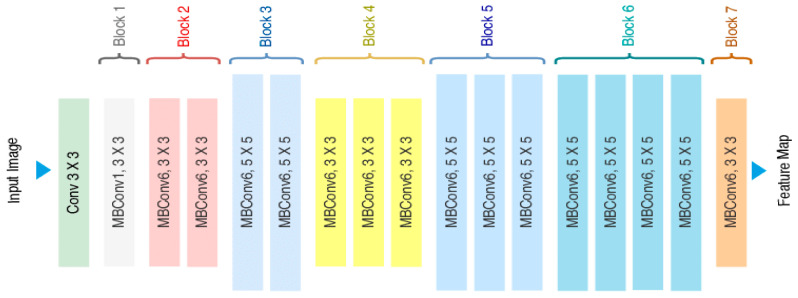
The 7 blocks of the efficient-net used in our model.

**Figure 7:**
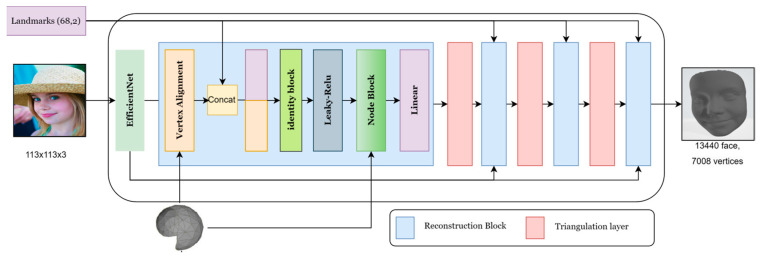
Architecture of our model generator.

Our model’s generator, based on [32], uses a single image and landmarks to create a 3D geometric model of the face. To extract the features from input images, we employ the widely used Efficient-Net [33] neural network with 7 blocks as the backbone, shown in Figure 6. Our generator is made up of four blocks called reconstruction blocks, each followed by a triangulation mesh layer, except for the final layer as shown in Figure 7. To recreate the first block, we combine the output from the 7 blocks with the semi-sphere and landmarks.

The Efficient-Net starts with a normal 2D 3 × 3 conv with padding 0 and stride of 1. Applying a 32-filter with a spatial extend of 3 yields a result of 111 × 111 × 32.

Mobile inverted bottleneck convolution (MBConvlayer) is a depthwise separable convolution where each block consists of a different number of layers with possible changes in dimensions. For MBConv1 and MBConv3 (3 × 3), we applied stride 1 and the same padding of 111 × 111 × 16 as block 2, and for MbConv6 (5 × 5), we applied stride 2, with the same padding of 57 × 57 × 24 output as block 3; for Block4, we get 29 × 29 × 80, and for the next blocks, 5, 6, and 7, we applied the same padding and stride as one but with different filters with each layer; thus, the final output features will be 29 × 29 × 320, which will be concatenated with semi-sphere landmarks as input for the first reconstruction block.

##### Reconstruction Block

Every reconstruction block includes two operations: vertex alignment [33] and the NODE block. The first operation, vertex alignment, aims to extract image features for vertices and combine them with the semi-sphere’s properties. Given a feature map, we compute a bilinearly interpolated image feature for each projected vertex point. We utilize the camera’s intrinsic matrix to project each vertex onto the image plane.

The semi-sphere comprises 141 vertices and 210 polygons, which will be projected onto the image. The output from vertex alignment, an image with the semi-sphere vertices projected on it, is concatenated with landmarks and fed into a leaky ReLU activation function.

**Figure 8:**
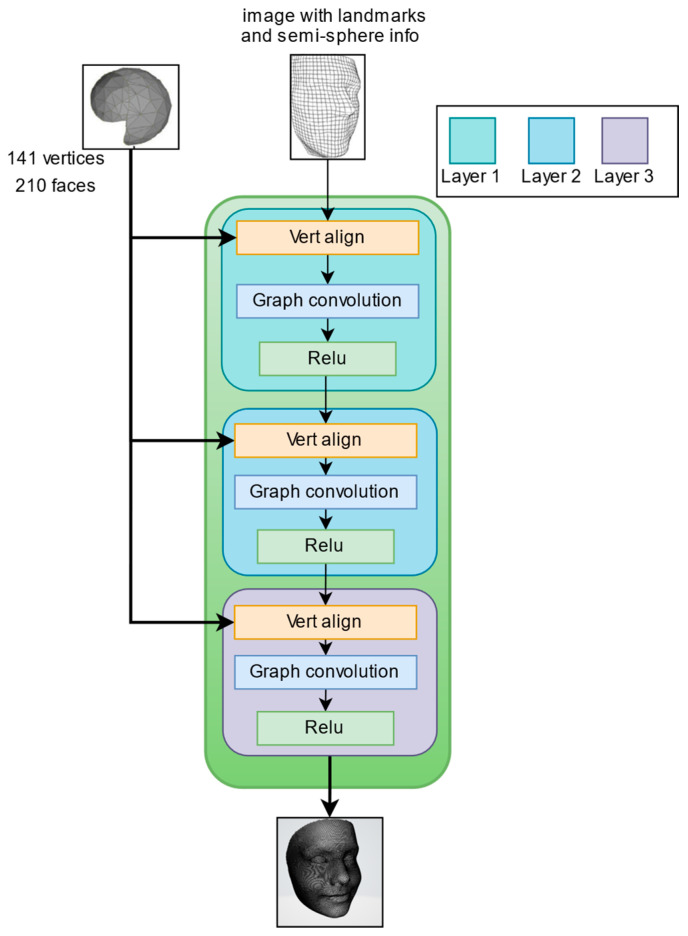
Node block architecture.

The second operation, the NODE block, is made up of three layers of graph convolution, each followed by a ReLU activation function, as illustrated in Figure 8.

The graph convolution [34] propagates information along mesh edges. For a set of input vertex features $\left(f_{i}\right)$ it computes updated features $\left(f_{i}^{\prime }\right)$.

$$f_{i}^{\prime }=leaky\;\mathrm{ReLU}\left(W_{0}f_{i}+\sum_{j\in N\left(i\right)}W_{1}f_{j}\right)$$

$N\left(i\right)$ represents the neighbor points of the (i)-th vertex in the mesh. $\left(W_{0}\right)$ and $\left(W_{1}\right)$ are learned weight matrices. This process allows each vertex to incorporate information from its neighboring vertices, effectively capturing the local structure of the mesh. The reconstruction node block employs multiple graph convolution layers to aggregate this information, enhancing the representation of the local mesh regions. The output of node block is then passed through the linear layer to the next block, which is the triangulation layer.

##### Triangulation Layer

Triangulation layers are used to turn the raw mesh into a finer mesh with more faces, vertices, and edges. The face can be reconstructed at ever-finer resolutions thanks to this refinement. This can be achieved by subdividing each triangular face of the mesh. Specifically, a vertex is inserted at the midpoint of each edge within a triangle, and new edges are drawn between these midpoints, effectively subdividing the original triangle into four smaller triangles. This recursive refinement process allows the mesh to capture finer geometric details and subtle variations in the surface, which is essential for applications requiring high-precision, realistic 3D modeling like ours. The increased density of the mesh enhances the accuracy of 3D reconstructions, enabling more detailed and lifelike representations.

#### 3.3.2. Discriminator Architecture

**Figure 9:**
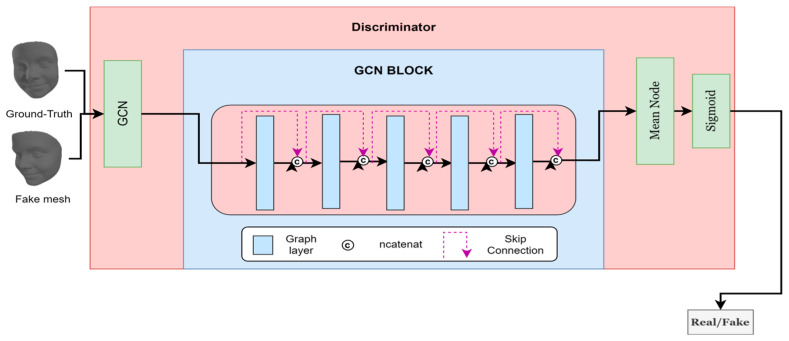
Architecture of our discriminator.

The discriminator architecture in our model, illustrated in Figure 9, is designed with a convolutional graph layer, followed by an advanced GCN block and a mean node function. The mean node function computes the average value of the features produced by the GCN block, which is crucial for effectively summarizing the feature information. Subsequently, a linear layer is applied to provide a binary classification outcome, distinguishing between authentic and synthetic (fake) meshes. The discriminator’s input is either a sample generated by the generator (fake) or a genuine sample (real) sourced from the dataset.

The GCN block, detailed in Figure 9 blue block, consists of five GCN layers. We incorporate connections linking the input and output of each layer through a concatenation operation, as shown in Figure 9. This architectural choice ensures that the model benefits from both shallow and deep feature representations. Each GCN layer outputs features with a dimension of 16, resulting in a cumulative output dimension of 96 for the entire GCN block.

This configuration is motivated by the need to capture intricate spatial relationships in the mesh data, leveraging the convolutional graph layer’s local feature extraction and the GCN block’s global feature aggregation. The mean node function plays a pivotal role in this architecture by effectively summarizing the node features, thus enhancing the subsequent linear classification layer’s performance. Empirical evaluations have demonstrated that this discriminator architecture significantly improves classification accuracy, thereby enhancing the overall robustness and stability of the GAN model.

#### 3.3.3. Datasets

We trained our network on the 300W-LP database [35], which contains 60,000 face images. We derived the real 3D meshes in 300W-LP using a standard optimization-based 3DMM fitting method. We used the ground truth 3D mesh to generate the face bounding box for each image before cropping and resizing it to 113 × 113 × 3 pixels. During training, we employ many data augmentation strategies, as indicated in algorithm 1, that have proven beneficial. We also evaluated our model using 300W-LP and AFLW2000-3D [36] which is a 3D dataset with 68 landmarks similar to 300W-LP but with 2000 images.

#### 3.3.4. Evaluation Metrics

We used many evaluation metrics for every step in the algorithm. To evaluate the best noise removal methods, we used the signal-to-noise ratio and structural similarity index. On the other hand, to evaluate the 3D construction, we used Chamfer Distance (CD) and Earth Mover’s Distance (EMD).

The Peak Signal-to-Noise Ratio (PSNR) is a widely used metric for evaluating the quality of reconstructed images, particularly in the context of image compression and restoration tasks. PSNR is derived from the mean squared error (MSE) between the original and reconstructed images, with higher values indicating better image quality. It is calculated in decibels (dB), providing an objective measure of the difference between the two images. Despite its simplicity and ease of computation, PSNR primarily focuses on pixel-wise differences, making it less sensitive to perceptual quality, especially when the human visual system’s characteristics are considered. PSNR is measured in decibels (dB) and is defined as follows:

(4) $$PSNR=10\times \log_{10}\left(\frac{L^{2}}{MSE}\right)$$

where L represents the maximum possible pixel value of the image and MSE is the Mean Squared Error between the original and reconstructed images.

The Structural Similarity Index (SSIM), on the other hand, offers a more holistic approach to image quality assessment by considering changes in structural information (s), luminance (L), and contrast (c). SSIM is designed to mimic the human visual system, providing a score between −1 and 1, where 1 indicates perfect similarity. Unlike PSNR, SSIM evaluates the structural similarity between the original and reconstructed images, making it more aligned with human perception. This makes SSIM particularly valuable for applications where visual quality is paramount. Our framework employs SSIM and PSNR metrics across different levels 0.1, 0.25, 0.5, and 0.75 to evaluate the efficacy of noise reduction. SSIM calculation is conducted using the following formula:

(5) $$SSIM=\left(l^{\alpha }\right)\times \left(c^{\beta }\right)\times \left(s^{\gamma }\right).$$

The weighting parameters α, β, and γ control the relative significance of each component. In most cases, the value of α, β, and γ are set to 1.

Chamfer Distance is a fundamental metric in the domain of 3D reconstruction. It quantifies the disparity between two-point clouds by considering the proximity of each point in the generated point cloud to the closest point in the ground truth point cloud, and vice versa. Specifically, for each point in one point cloud, the Chamfer Distance identifies the nearest point in the other point cloud and computes the squared Euclidean distance. The sum of these distances over all points provides a comprehensive measure of similarity between the two-point clouds. In practice, Chamfer Distance is employed to guide the generator in producing point clouds that closely resemble the ground truth, ensuring high fidelity in 3D reconstruction tasks. The Chamfer Distance DCD between two point clouds *P* and *Q* is defined as follows:

(6) $$D_{\mathrm{CD}}\left(P,Q\right)=\sum_{p\in P}\underset{q\in Q}{\min}|p-q{|}^{2}+\sum_{q\in Q}\underset{p\in P}{\min}|q-p{|}^{2}.$$

The Earth Mover’s Distance (EMD) is a robust metric for comparing two probability distributions, often represented as point clouds or histograms. In the context of 3D reconstruction, EMD measures the minimum amount of work required to transform one point cloud into another, where work is defined as the product of the number of points moved and the distance they are moved. This metric effectively captures the similarity between two-point clouds by considering not only the distances between points but also the distribution of mass across the clouds. The Earth Mover’s Distance DEMD between two point clouds *P* and *Q* is calculated as follows:

(7) $$D_{\mathrm{EMD}}\left(P,Q\right)=\underset{\phi :P\to Q}{\min}\sum_{p\in P}|p-\phi \left(p\right)|.$$

where ϕ represents a bijection (a one-to-one and onto mapping) between two-point clouds *P* and *Q*.

## 4. Results

The following section summarizes and analyzes the results from each stage of the proposed framework. In Section 4.1, we present the results of a comparison of several methods for reducing noise from face images. The comparison is carried out using PSNR and SSIM. In Section 4.2, we show the outcomes of creating a 3D mesh model from a single 2D image.

### 4.1. Result of Noise Removal

Table 1 presents the outcomes of noise reduction using various deep learning frameworks trained for 200 epochs, employing a learning rate of 1 × 10^-5^ and the Adamax optimizer. The comparison is conducted across four distinct noise levels, 0.10, 0.25, 0.50, and 0.75. The results demonstrate that the U-shaped CNN combined with the MD model outperforms other deep learning models in effectively removing noise at different levels.

The improvements in PSNR and SSIM values demonstrated by the U-shaped CNN with matrix factorization have significant practical implications for 3D face reconstruction. Higher PSNR values indicate a better signal-to-noise ratio, which translates to clearer, more detailed images. This is crucial for accurately capturing facial features and expressions. The SSIM improvements suggest that the structural information of the face is better preserved, which is essential for maintaining the integrity of facial geometry during reconstruction.

The model’s robust performance across various noise levels, particularly in high-noise scenarios, is particularly valuable. In real-world applications, input images often suffer from varying degrees of noise due to factors like poor lighting conditions or low-quality cameras. The ability to effectively denoise images at different noise levels (0.1, 0.25, 0.5, and 0.75) ensures that our 3D reconstruction pipeline can handle a wide range of input quality. This versatility is crucial for practical deployments where image quality cannot always be controlled.

At lower noise levels (0.1 and 0.25), the improvements in PSNR and SSIM contribute to capturing fine details and subtle facial features. As noise levels increase (0.5 and 0.75), the model’s superior performance becomes even more critical, allowing for accurate reconstruction even from severely degraded inputs. This robustness to high noise levels expands the usability of our 3D reconstruction system in challenging environments or with low-quality imaging devices.

### 4.2. Result of 3D Reconstruction

In this section, we present the results of our model using both qualitative and quantitative evaluations. We trained our GAN model using the 300W-LP dataset and exclusively evaluated it using the AFLW2000-3D dataset. To reduce computational requirements, we lowered the dimensions of the generator’s input image to 113 × 113 pixels and the features of the 3D mesh to 13,440 faces and 6910 vertices. Table 2 outlines the hardware used in this process.

Our model is compared quantitatively using Earth Mover’s Distance and Chamfer Distance to recent research on 3D face prediction from a single image, including Deep3D [19], GAN2- Shape [21], Deca [6], hybrid-level contextual information-based model (HLCI) [37], and 3DDFA [38]. Table 3 presents a quantitative comparison of the models, demonstrating that our proposed model achieves state-of-the-art performance. Specifically, on the 300W-LP dataset, our model improves the Chamfer Distance (CD) by 0.0075 and the Earth Mover’s Distance (EMD) by 0.120. Furthermore, on the AFLW2000 dataset, our model achieves improvements of 0.057 in CD and 0.28 in EMD.

**Figure 10:**
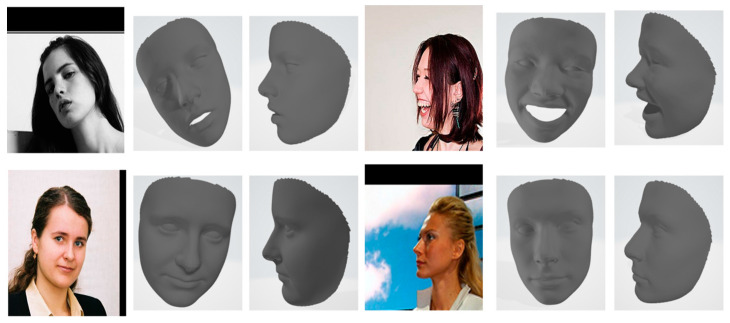
Reconstruction of our model for different face poses and lighting.

**Figure 11:**
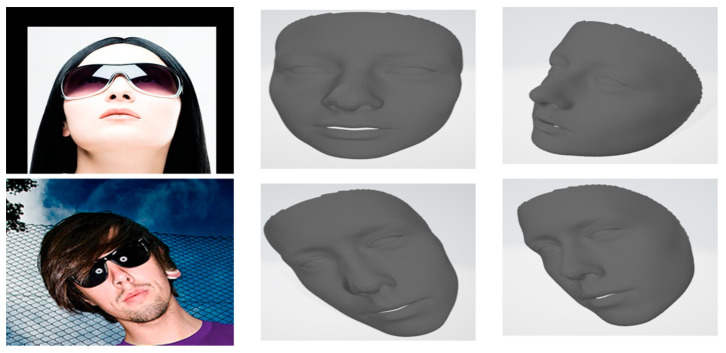
Reconstruction of our model for images with partial facial occlusions.

**Figure 12:**
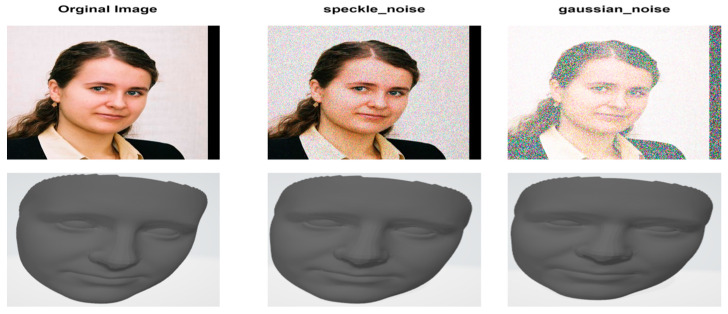
Reconstruction of our model for an image with noise.

Our model demonstrates superior robustness to variations in head positioning, occlusion, noise, and lighting conditions. This is evidenced by the consistently low CD and EMD scores across the diverse AFLW2000-3D dataset, which includes a wide range of challenging scenarios. The model accurately reconstructs faces at various angles, as seen in Figure 10. Our method maintains performance even with partial facial occlusions as shown in Figure 11, thanks to the effective use of landmarks and the GCN architecture. The preprocessing stage, particularly the U-shaped CNN with MD, significantly reduces input noise, allowing for accurate reconstruction even from noisy images, as shown in Figure 12.

As shown in Table 3, for the 300W-LP dataset, our model reduced CD by 0.0075 and EMD by 0.120 compared to the next-best method. These improvements translate to more accurate facial geometry reconstructions, which is crucial for applications such as facial recognition, virtual try-on systems, and computer graphics in film and gaming industries. Lower CD values indicate that the reconstructed 3D points are closer to the ground truth, resulting in more faithful representation of facial features. The reduction in EMD suggests that the overall distribution of points in the reconstructed face better matches the ground truth, leading to more natural-looking 3D models.

For the AFLW2000-3D dataset, our model achieved improvements of 0.057 in CD and 0.28 in EMD. While these improvements are substantial, the difference in magnitude compared to the 300W-LP results can be attributed to the distinct characteristics of each dataset. The 300W-LP dataset contains a wider range of poses and expressions, which our model seems to handle particularly well. This suggests that our approach is especially effective in capturing and reconstructing diverse facial orientations and expressions.

The AFLW2000-3D dataset, while smaller, includes more challenging real-world images. The improvements on this dataset demonstrate our model’s robustness to real-world variability in lighting, resolution, and facial appearances. This is particularly important for practical applications where input images may not be captured under controlled conditions.

**Figure 13:**
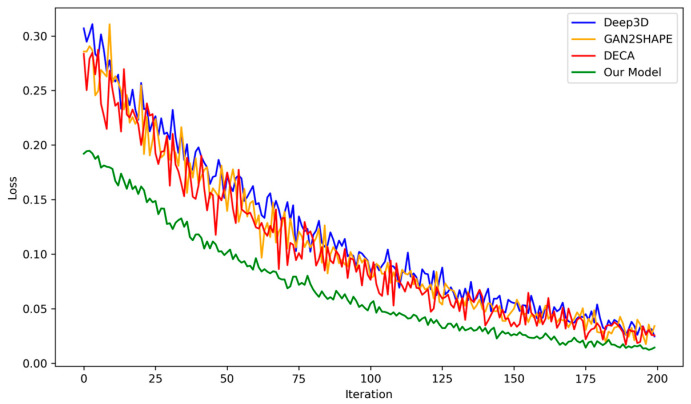
Comparison of different model losses for 200 epochs.

Figure 13 presents a comparative analysis of the loss values of our model against the Deep3d, the GAN2SHAPE, and DECA models. From the visualization, it is evident that the modifications implemented in our model not only result in lower loss but also enhance stability compared to the other models, which exhibit significant oscillations in loss. These oscillations can be indicative of vanishing gradients, mode collapse, or non-convergence in GANs. Achieving stability in the GAN model enables prolonged training, thereby yielding better results without the issue of mode collapse. Our model demonstrates success in stabilizing the training process. These stability improvements not only enhance the model’s performance but also make it more viable for large-scale, resource-efficient deployments in various 3D face reconstruction applications.

**Figure 14:**
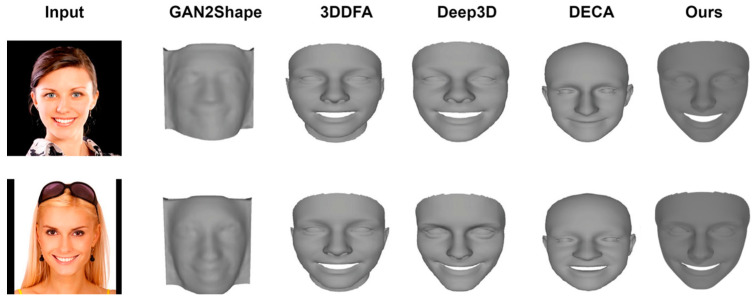
Qualitative comparison between our model and other methods.

Figure 14 presents a qualitative comparison between the proposed model and other state-of-the-art methods for 3D face reconstruction. The figure showcases the results of different approaches applied to the same input images, allowing for a visual assessment of the reconstruction quality. Our model demonstrates superior performance in capturing fine facial details, accurate geometry, and natural expressions compared to the other methods shown. This visual comparison complements the quantitative results presented in Table 3, providing a more intuitive understanding of the improvements achieved by our method.

### 4.3. Effectiveness of GCN-Based Architecture

To demonstrate the effectiveness of our GCN-based architecture, we conducted an ablation study comparing our full model against a version without the GCN components. Table 4 presents the quantitative results of this comparison.

**Figure 15:**
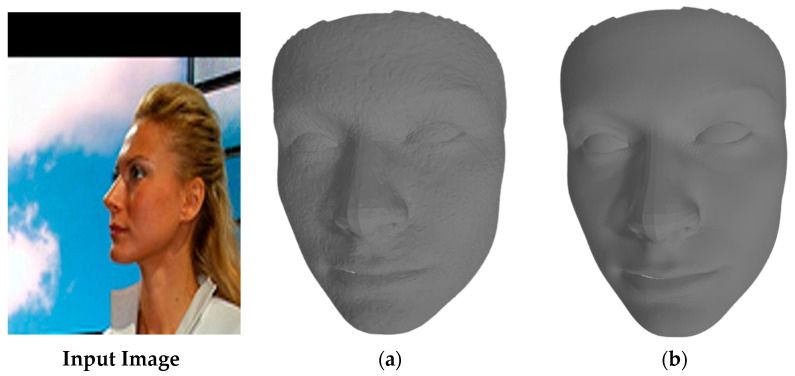
Visual demonstration of the impact of GCN-based architecture. The full model (**a**) produces the most accurate and detailed reconstructions, particularly in facial feature alignment and surface detail, compared to (**b**) the model without GCN.

As shown in Table 4, the incorporation of the GCN-based architecture leads to a significant improvement in both Chamfer Distance and Earth Mover’s Distance metrics. The GCN components allow for better capture of local and global facial geometries, resulting in more accurate 3D reconstructions; this also shown in in Figure 15.

## 5. Conclusions

In this paper, we have introduced an advanced method for 3D face reconstruction from single 2D images, leveraging the strengths of modified adversarial neural networks and graph neural networks. Our innovative approach effectively addresses common challenges such as mode collapse and instability in GAN training by incorporating a novel generator architecture based on Graph Convolutional Networks (GCNs), along with a new loss function and identity blocks. The integration of landmarks enhances the recognition process, while the non-parametric Efficient-Net decoder captures critical facial geometry features. Our lightweight discriminator further improves the accuracy and stability of the generated 3D facial models.

Empirical evaluations on the 300W-LP and AFLW2000-3D datasets demonstrate that our model significantly outperforms existing state-of-the-art methods. Specifically, on the 300W-LP dataset, our approach reduces the Chamfer Distance (CD) by 62.7% (from 0.0201 to 0.0075) and Earth Mover’s Distance (EMD) by 57.1% (from 0.280 to 0.120) compared to the previous best method (DECA). On the AFLW2000-3D dataset, while our CD (0.0573) is slightly higher than that of DECA (0.0150), we achieved a substantial 582.9% improvement in EMD (from 0.0410 to 0.280), indicating better overall shape fidelity.

While our study demonstrates significant improvements in 3D face reconstruction accuracy and stability, future work could explore additional metrics for evaluating the diversity and fidelity of the generated 3D models. Although traditional GAN evaluation metrics like Inception Score or Fréchet Inception Distance may not be directly applicable to our conditional GAN setup, developing specialized metrics for 3D face reconstruction could provide further insights into the model’s performance across a wide range of facial variations.

Overall, our proposed framework represents a significant advancement in the field of 3D face reconstruction, offering a robust and efficient solution for generating high-fidelity 3D facial models from single 2D images. Future work may explore the integration of additional features and further optimizations using evolutionary algorithms to enhance the performance and applicability of our method in various real-world scenarios.

## Figures and Tables

**Table 1:** Result from removing noise using different models.

Method	PSNR				SSIM			
	0.1	0.25	0.5	0.75	0.1	0.25	0.5	0.75
Original Image	18.31	17.31	12.34	8.34	81.31%	70.20%	43.23%	35.81%
CNN with attention	23.9	22.85	26.89	25.02	87.99%	82.96%	79.67%	72.06%
VAEs	31.8	30.96	28.19	24.87	89.12%	85.36%	82.9%	79.18%
Auto-encoder	30.7	28.81	25.43	24.98	81.25%	76.42%	73.9%	72.08%
D-U-NET	38.4	36.14	35.42	26.77	94.80%	93.03%	91.21%	83.32%
U-shaped CNN with MD	41.29	40.10	34.10	28.80	95.98%	94.04%	92.7%	86.09%

**Table 2:** Hardware specifications.

Device	Description
Processors	Intel (R) Core (TM) i7-10750H CPU @ 2.60 GHz
RAM	16.0 GB
GPU	NVIDIA GeForce RTX 3050Ti
HD Space	Samsung SSD 970 EVO Plus 2TB

**Table 3:** Comparison between our method and four state-of-the-art methods.

Methods	300W-LP		AFLW2000-3D	
	CD	EMD	CD	EMD
GAN2Shape	0.108	0.900	0.1127	0.1440
3DDFA	0.091	0.810	0.0195	0.2800
Deep3D	0.0263	0.210	0.3300	0.1600
DECA	0.0201	0.2800	0.0150	0.0410
HLCI	0.350	0.3100	0.0981	0.1810
Ours	0.00750	0.1200	0.0573	0.2800

**Table 4:** Ablation study results for GCN-based architecture.

Model	300W-LP		AFLW2000-3D	
	CD	EMD	CD	EMD
With GCN (Full Model)	0.00750	0.1200	0.0573	0.2800
Without GCN	0.0853	0.1480	0.0670	0.3140

## Data Availability

The data that support the findings of this study are available on request from the corresponding author.

## References

[1] Blanz V., Vetter T. A morphable model for the synthesis of 3D faces. Proceedings of the 26th Annual Conference on Computer Graphics and Interactive Techniques, SIGGRAPH 1999.

[2] Roth J., Tong Y., Liu X. (2017). Adaptive 3D Face Reconstruction from Unconstrained Photo Collections. IEEE Trans. Pattern Anal. Mach. Intell..

[3] Feng Y., Wu F., Shao X., Wang Y., Zhou X. (2018). Joint 3d face reconstruction and dense alignment with position map regression network. Lecture Notes in Computer Science (Including Subseries Lecture Notes in Artificial Intelligence and Lecture Notes in Bioinformatics).

[4] Ichim A.E., Bouaziz S., Pauly M. (2015). Dynamic 3D avatar creation from hand-held video input. ACM Trans. Graph..

[5] Dargan S., Kumar M. (2020). A comprehensive survey on the biometric recognition systems based on physiological and behavioral modalities. Expert. Syst. Appl..

[6] Feng Y., Feng H., Black M.J., Bolkart T. (2021). Learning an animatable detailed 3D face model from in-the-wild images. ACM Trans. Graph..

[7] Gecer B., Ploumpis S., Kotsia I., Zafeiriou S. Ganfit: Generative adversarial network fitting for high fidelity 3D face reconstruction. Proceedings of the IEEE Computer Society Conference on Computer Vision and Pattern Recognition.

[8] Deng Y., Yang J., Xu S., Chen D., Jia Y., Tong X. Accurate 3D face reconstruction with weakly-supervised learning: From single image to image set. Proceedings of the IEEE Computer Society Conference on Computer Vision and Pattern Recognition Workshops.

[9] Scarselli F., Gori M., Tsoi A.C., Hagenbuchner M., Monfardini G. (2009). The graph neural network model. IEEE Trans. Neural Netw..

[10] Defferrard M., Bresson X., Vandergheynst P. (2016). Convolutional neural networks on graphs with fast localized spectral filtering. Advances in Neural Information Processing Systems.

[11] Booth J., Roussos A., Zafeiriou S., Ponniahy A., Dunaway D. A 3D morphable model learnt from 10,000 faces. Proceedings of the IEEE Computer Society Conference on Computer Vision and Pattern Recognition.

[12] Yang D., Hong S., Jang Y., Zhao T., Lee H. Diversity-sensitive conditional generative adversarial networks. Proceedings of the 7th International Conference on Learning Representations, ICLR 2019.

[13] Zhou P., Xie L., Ni B., Tian Q. (2022). Searching Towards Class-Aware Generators for Conditional Generative Adversarial Networks. IEEE Signal Process Lett..

[14] Pasini M.L., Yin J. (2023). Stable parallel training of Wasserstein conditional generative adversarial neural networks. J. Supercomput..

[15] Fathallah M., Sakr M., Eletriby S. (2023). Stabilizing and Improving Training of Generative Adversarial Networks Through Identity Blocks and Modified Loss Function. IEEE Access.

[16] Deng Q., Ma L., Jin A., Bi H., Le B.H., Deng Z. (2022). Plausible 3D Face Wrinkle Generation Using Variational Autoencoders. IEEE Trans. Vis. Comput. Graph..

[17] Tran A.T., Hassner T., Masi I., Medioni G. Regressing robust and discriminative 3D morphable models with a very deep neural network. Proceedings of the 30th IEEE Conference on Computer Vision and Pattern Recognition, CVPR 2017.

[18] Richardson E., Sela M., Kimmel R. 3D face reconstruction by learning from synthetic data. Proceedings of the 2016 4th International Conference on 3D Vision, 3DV 2016.

[19] Lin J., Yuan Y., Shao T., Zhou K. Towards high-fidelity 3d face reconstruction from in-the-wild images using graph convolutional networks. Proceedings of the IEEE/CVF Conference on Computer Vision and Pattern Recognition.

[20] Nikolentzos G., Vazirgiannis M. (2020). Random walk graph neural networks. Adv. Neural Inf. Process. Syst..

[21] Pan X., Dai B., Liu Z., Loy C.C., Luo P. Do 2D Gans Know 3D Shape? Unsupervised 3D Shape Reconstruction from 2D Image Gans. Proceedings of the ICLR 2021—9th International Conference on Learning Representations.

[22] Schroff F., Kalenichenko D., Philbin J. FaceNet: A unified embedding for face recognition and clustering. Proceedings of the IEEE Computer Society Conference on Computer Vision and Pattern Recognition.

[23] Cheng S., Tzimiropoulos G., Shen J., Pantic M. (2021). Faster, Better and More Detailed: 3D Face Reconstruction with Graph Convolutional Networks. Lecture Notes in Computer Science (Including Subseries Lecture Notes in Artificial Intelligence and Lecture Notes in Bioinformatics).

[24] Sengupta S., Lichy D., Kanazawa A., Castillo C.D., Jacobs D.W. (2022). SfSNet: Learning Shape, Reflectance and Illuminance of Faces in the Wild. IEEE Trans. Pattern Anal. Mach. Intell..

[25] Deng Z., Liang Y., Pan J., Liao J., Hao Y., Wen X. (2023). Fast 3D face reconstruction from a single image combining attention mechanism and graph convolutional network. Vis. Comput..

[26] Zhou Y., Deng J., Kotsia I., Zafeiriou S. Dense 3D face decoding over 2500FPS: Joint texture & shape convolutional mesh decoders. Proceedings of the IEEE Computer Society Conference on Computer Vision and Pattern Recognition.

[27] Yang L., Zhang Z., Song Y., Hong S., Xu R., Zhao Y., Zhang W., Cui B., Yang M.-H. (2023). Diffusion Models: A Comprehensive Survey of Methods and Applications. ACM Comput. Surv..

[28] Rombach R., Blattmann A., Lorenz D., Esser P., Ommer B. High-Resolution Image Synthesis with Latent Diffusion Models. Proceedings of the IEEE Computer Society Conference on Computer Vision and Pattern Recognition.

[29] Huang Z., Chan K.C., Jiang Y., Liu Z. Collaborative Diffusion for Multi-Modal Face Generation and Editing. Proceedings of the IEEE Computer Society Conference on Computer Vision and Pattern Recognition.

[30] Salimans T., Goodfellow I., Zaremba W., Cheung V., Radford A., Chen X. (2016). Improved techniques for training GANs. Advances in Neural Information Processing Systems.

[31] Fathallah M., Sakr M., Eletriby S. (2023). Novel Framework for Generating Criminals Images Based on Textual Data Using Identity GANs. Comput. Mater. Contin..

[32] Wang N., Zhang Y., Li Z. Pixel2Mesh—Generating Meshes from Single RGB Images. Proceedings of the European Conference on Computer Vision (ECCV).

[33] Tan M., Le Q.V. EfficientNet: Rethinking model scaling for convolutional neural networks. Proceedings of the 36th International Conference on Machine Learning, ICML 2019.

[34] Kipf T.N., Welling M. Semi-supervised classification with graph convolutional networks. Proceedings of the 5th International Conference on Learning Representations, ICLR 2017.

[35] Zhu X., Liu X., Lei Z., Li S.Z. (2019). Face Alignment in Full Pose Range: A 3D Total Solution. IEEE Trans. Pattern Anal. Mach. Intell..

[36] Kostinger M., Wohlhart P., Roth P.M., Bischof H. Annotated facial landmarks in the wild: A large-scale, real-world database for facial landmark localization. Proceedings of the IEEE International Conference on Computer Vision.

[37] Liu Y., Ran T., Yuan L., Lv K., Zheng G. (2024). 3D face reconstruction from a single image based on hybrid-level contextual information with weak supervision. Comput. Graph..

[38] Niu C., Nan F., Wang X. (2021). A super resolution frontal face generation model based on 3DDFA and CBAM. Displays.

